# ‘GP services are still heteronormative’: Sexual minority cisgender women’s experiences of UK menopause healthcare – Health equity implications

**DOI:** 10.1177/20533691241279887

**Published:** 2024-09-09

**Authors:** Sue Westwood

**Affiliations:** 1 York Law School, 8748 University of York, York, UK

**Keywords:** Lesbian, gay, bisexual, pansexual, queer women, menopause, heteronormative healthcare, sexual identity, health equity

## Abstract

**Objective:**

This article reports on UK sexual minority cisgender women’s experiences of menopause health and healthcare, based on a data subset from a study exploring lesbian, gay, bisexual, and queer (LGBTQ+) menopause.

**Methods:**

An online survey was conducted with UK LGBTQ + individuals who went through/are going through the menopause. Quantitative data were analysed using simple descriptive statistics. Qualitative data were analysed using thematic analysis.

**Results:**

Cisgender respondents comprised 51 lesbian, gay, bisexual, pansexual, queer, and ‘other’ women, aged between 17 and 89 years. They reported similar types and levels of menopause symptoms as heterosexual cisgender women in other studies, apart from higher levels of anxiety and depression, especially bisexual women. Dissatisfaction regarding menopause healthcare services related to access, information, and heteronormative/heterosexist provision.

**Conclusions:**

Healthcare providers must ensure they provide inclusive menopause services to sexual minority cisgender women.

## Introduction

There is growing awareness about the menopause and the need for effective menopause healthcare.^1–5^ The menopause – when a woman’s periods stop and she is no longer able to reproduce – is preceded by perimenopause (before periods cease) and followed by post-menopause (12 months afterwards). Most women experience at least one symptom, and often several, including difficulties with memory and concentration; night sweats; hot flushes; anxiety/depression; erratic periods and/or flooding; reduced/loss of libido; recurrent urinary tract infections; and disturbed sleep.^6,7^ Some gender non-binary individuals and transgender men are also affected.^8^

Most menopause literature presumes menopause to be the experience of heterosexual cisgender women. There is a paucity of research about sexual minority cisgender (SMC) women’s menopause experiences and healthcare needs.^2,3,9^ None of the major recent UK menopause studies analysed their data by sexual identity.^6,7,10,11^ This is a significant knowledge gap, especially given a recent 27-country study found that 11% of the population surveyed identified as being attracted to the same/both sexes.^12^ SMC women’s menopause experiences are likely to be impacted by their stigmatised minority sexual identities.^13^ They generally have poorer physical and mental health and higher levels of dissatisfaction with healthcare than heterosexual cisgender women.^14,15^ A key barrier encountered by SMC women relates to sexual identity disclosure.^16^ Many SMC women, especially older women, choose not to disclose their sexual identities in healthcare contexts, to avoid potential/actual prejudice and discrimination. This creates particular challenges when seeking medical advice on reproductive issues.^17^

SMC women are entitled to equitable healthcare. Under the UK Equality Act 2010, sexual orientation is protected from discrimination, including in the provision of goods and services such as healthcare. NICE menopause guidance^18^ states there should be ‘an individualised approach at all stages of diagnosis, investigation and management of perimenopause and menopause’ (Para 1). The UK Government’s LGBT Action Plan^19^ states

We will ensure that LGBT people’s needs are at the heart of the National Health Service. We want LGBT people to easily access healthcare when they need it most and feel comfortable disclosing their sexual orientation or gender identity so that they get the best possible care (2).

This article reports on a sample of 51 lesbian, gay, bisexual, pansexual, and queer (‘sexual minority’) cisgender (SMC) women who responded to a UK survey about LGBTQ + menopause. Its aim is to describe their health and healthcare experiences, analyse their dissatisfactions, and propose ways forward.

## Methods

This project was approved by the University of York’s Economics, Law, Management, Politics and Sociology (ELMPS) Ethics Committee. UK LGBTQ + individuals who experienced/are currently experiencing the menopause were surveyed. Respondents were recruited via professional networks, menopause advocacy and LGBTQ + advocacy organisations, and social media (Twitter and Facebook). Simple descriptive statistics were used to analyse qualitative data; qualitative data were coded, anonymised, and analysed using thematic analysis.^20^ Full details of methods, including the questionnaire, recruitment, and data analysis, are reported elsewhere.^21^

Of 66 respondents, 51 were lesbian, gay, bisexual, pansexual, queer, and ‘other’ cisgender women. The remaining respondents were transgender and gender non-binary individuals, the results for whom, analysed by gender identity, are reported elsewhere.^22^

## Results

### Quantitative results from the survey

Demographic data are summarised in Table 1.

**Table 1.** Respondent demographics.

	Lesbian	Gay	Bisexual	Pansexual	Queer	Other^a^	Total
Sexual identity	27 (53%)	2 (4%)	15 (29%)	2 (4%)	2 (4%)	3 (6%)	51
Age band							
18–29	3	0	1	0	0	0	4
30–39	2	0	1	0	1	0	4
40–49	8	0	9	1	0	1	19
50–59	6	1	2		1	2	12
60–69	6	1	2	1	0	0	10
70–79	1	0	0	0	0	0	1
80–89	1	0	0	0	0	0	1
Ethnicity							
White (English, Welsh, Scottish, Northern Irish, British, Irish, Gypsy or Irish Traveller, Roma, any other White background)	27	2	15	2	2	3	51 (100%)
Disability							
No disability	19	1	5	2	1	1	29 (57%)
Disability	8	1	10	0	1	2	22 (43%)
Currently experiencing/previously experienced menopause							
Currently experiencing menopause	12	1	12	0	2	2	29 (57%)
Previously experienced menopause	15	1	3	2	0	1	22 (43%)
Current/most recent occupation							
Manager, director, or senior official	6	1	2	0	0	1	10 (20%)
Professional occupation (degree or equivalent, sometimes with postgraduate qualifications and/or formal period of experience-related training)	13	0	9	1	2	2	27 (53%)
Associate professional occupations (high-level vocational qualification, often involving substantial full-time training)	2	0	0	0	0	0	2 (4%)
Administrative and secretarial occupations	2	1	2	1	0	0	6 (12%)
Skilled trades occupations	1	0	0	0	0	0	1 (2%)
Caring, leisure, and other service occupations	1	0	0	0	0	0	1 (2%)
Sales and customer service occupations	1	0	0	0	0	0	1 (2%)
Process, plant, and machine operatives	0	0	0	0	0	0	0
Elementary occupations (requiring minimum general level of training/education)	1	0	0	0	0	0	1 (2%)
Long-term unemployed	0	0	1	0	0	0	1 (2%)

^a^Other = ‘Asexual/lesbian’, ‘Pansexual ARO [aromantic] and ACE [asexual]’, and ‘Queer/neuroqueer/pansexual’.

Respondents’ age of onset and symptoms are summarised in Table 2.

**Table 2.** Reported age of onset and symptoms.

	Lesbian (*n*-27)	Gay (*n*-2)	Bisexual (*n* = 15)	Pansexual (*n*-2)	Queer (*n*-2)	Other^a^ (*n* = 3)	Total (*n* = 51)
Age of onset							
18–29	0	0	0	0	0	0	0
30–39	1	0	4	0	1	1	7 (14%)
40–49	13	2	8	2	1	1	27 (53%)
50–59	8	0	2	0	0	1	11 (26%)
Don’t know/not sure/unclear	5	0	1	0	0	0	5 (10%)
*Total*	27	2	15	2	2	3	51
Symptoms							
Difficulty sleeping	22	0	13	2	2	3	42 (82%)
Anxiety and/or depression	21	1	15	1	2	2	42 (82%)
Hot flushes	21	2	12	2	2	1	40 (78%)
Problems with memory/concentration	19	2	15	2	2	3	43 (84%)
Night sweats	17	2	11	1	2	1	34 (67%)
Joint stiffness or aches	15	1	11	1	1	3	32 (63%)
Reduced/loss of libido	16	1	9	0	2	2	30 (58%)
Other changes to mood	12	0	7	0	1	3	23 (45%)
Headaches	10	0	7	0	1	2	20 (39%)
Palpitations	9	0	7	1	0	2	19 (37%)
Vaginal itching/dryness	11	1	5	1	1	1	20 (39%)
Irregular/excessive bleeding	5	1	6	1	1	1	15 (29%)
Recurrent urinary tract infections	4	1	1	0	0	1	7 (14%)

^a^Other = ‘Asexual/lesbian’, ‘Pansexual ARO [aromantic] and ACE [asexual]’, and ‘Queer/neuroqueer/pansexual’.

A total of 45 (88%) respondents reported seeking General Practitioner (GP) help in relation to the menopause. Their reasons for seeking help are summarised in Table 3.

**Table 3.** Reasons for consulting GP about menopause.

Symptom:	Lesbian (*n*-22)	Gay (*n*-2)	Bisexual (*n* = 14)	Pansexual (*n*-2)	Queer (*n*-2)	Other^a^ (*n* = 3)	Total (*n* = 45)
Difficulty sleeping	12	0	10	0	2	3	27 (60%)
Problems with memory/concentration	11	2	8	1	2	3	27 (60%)
Anxiety and/or depression	9	1	11	1	2	1	25 (56%)
Hot flushes	9	2	8	0	1	1	21 (47%)
Night sweats	9	2	6	0	1	1	19 (42%)
Joint stiffness or aches	5	0	7	1	1	1	15 (33%)
Other changes to mood	7	0	5	0	0	2	14 (31%)
Headaches	7	0	4	0	0	2	13 (29%)
Irregular/excessive bleeding	7	1	4	0	0	0	12 (27%)
Vaginal itching/dryness	7	1	2	0	1	0	11 (24%)
Reduced/loss of libido	6	1	3	0	0	1	11 (24%)
Palpitations	4	0	4	1	0	1	10 (22%)
Recurrent urinary tract infections	2	1	1	0	0	0	4 (9%)

^a^Other = ‘Asexual/lesbian’, ‘Pansexual ARO [aromantic] and ACE [asexual]’, and ‘Queer/neuroqueer/pansexual’.

**Figure 1.**
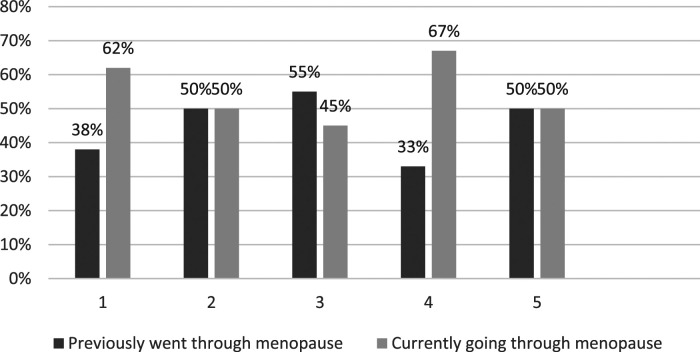
GP menopause consultation: ‘Do you feel your GP listened closely to your concerns?’ (*n* = 45). 1 (Very Satisfied)–5 (Very Dissatisfied). % is of total for each rating.

**Figure 2.**
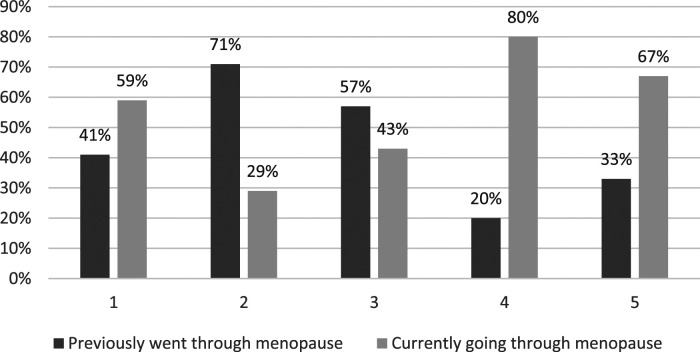
GP menopause consultation: ‘Do you feel your GP took your concerns seriously?’ (*n* = 45). 1 (Very Satisfied) 5 (Very Dissatisfied). % is of total for each rating.

**Figure 3.**
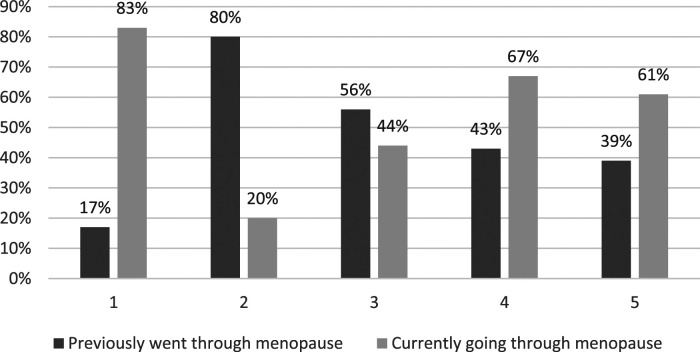
GP menopause consultation: ‘Did you receive sufficient guidance and information from your GP? (*n* = 45). 1 (Very Satisfied)–5 (Very Dissatisfied). % is of total for each rating.

**Figure 4.**
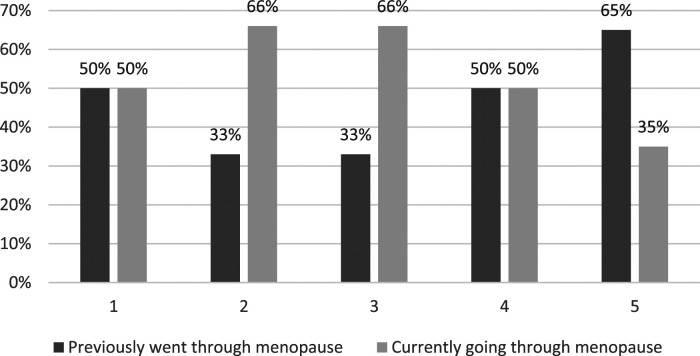
GP menopause consultation: ‘Overall, how satisfied are you with the menopause support you GP is providing/provided?’ (*n* = 45). 1 (Very Satisfied)–5 (Very Dissatisfied). % is of total for each rating.

Respondents’ satisfaction levels with GP support in relation to the menopause are depicted in Figures 1 to 4.

### Key quantitative results

• All but one (99%) of the respondents reported at least one menopause-related symptom; two-thirds (67%) reported seven to ten symptoms. Most frequently reported symptoms were problems with memory/concentration (84%); difficulty sleeping (82%); anxiety and/or depression (82%); hot flushes (78%); and night sweats (67%).
• Compared with lesbians, a greater proportion (all) of the bisexual women reported problems with anxiety and/or depression (15 [100%] bisexual cisgender women; 21 [78%] cisgender lesbians) and memory/concentration (15 [100%] bisexual cisgender women; 19 [70%] cisgender lesbians).
• A total of 45 (88%) respondents reported seeking General Practitioner (GP) help in relation to menopause symptoms, the most frequent concerns being difficulty sleeping (60%); problems with memory/concentration (60%); anxiety and/or depression (56%); hot flushes (47%); and night sweats (42%). A total of 27 (60%) respondents reported raising concerns about four or more symptoms with their GP.
• Almost half of respondents (24 = 47%) were slightly or very dissatisfied with GP menopause support. Over half of these women (13 = 54%) were currently going through the menopause. A third (17 = 33%) of respondents were very dissatisfied, a third of whom (6 = 33%) were currently going through the menopause. The primary concern related to the provision of information and guidance.

### Qualitative results from the survey

Respondents’ free-text responses indicated there were five main areas of dissatisfaction with healthcare providers: access issues; feeling menopause symptoms were dismissed/discounted; issues with navigating minority sexualities; heteronormative assumptions among healthcare professionals; heterosexist menopause information; and guidance. A sample of comments in relation to each is outlined below.

#### Access issues

Concerns were raised about accessing an appropriate healthcare professional. For example,

*My GP was male and told me to see a female GP in the future as he didn’t like to deal with ‘women’s problems’!* (SR23, 40–49, cisgender lesbian, currently going through menopause).

*Difficult to get appointments as only one GP does ‘women’s’ health’ and she is part-time* (SR60, 50–59, queer cisgender woman, currently going through menopause).

*Given my knowledge in menopause my GP was willing to listen, and it was a two-way discussion. The most frustrating thing was that she initially assumed I was heterosexual and then getting to see her consistently was very difficult due to her working patterns* (SR36, 50–59, gay cisgender woman, currently going through menopause).

*I tried to speak to a GP, but the appointment I was given was with a nurse… I felt dismissed and not listened to. I was told to use an app called Balance, but other than that no support at all. Very alienating experience* (SR13, 40–49, bisexual cisgender woman, currently going through menopause).

### Feeling menopause symptoms were dismissed/discounted

Concerns were raised by respondents about feeling their symptoms were dismissed and/or discounted. For example,

*I felt very lost and confused when I was told I was menopausal after years of feeling like I was going mad. I will no longer see a male GP in regard to the menopause as I had such a bad experience previously. I felt humiliated and that I was making a fuss, and the menopause isn't an issue* (SR23, *40*–49, cisgender lesbian, currently going through menopause).

*It took over 2 years for my GP to agree to testing my hormone levels as they insisted I was too young to be menopausal. When they eventually agreed I was found to be post-menopausal… I have been offered no further testing, particularly in relation to current hormone levels and whether the HRT is working… I have been repeatedly fobbed off by my GP practice. I have not been offered anything additional to the HRT e.g. counselling, support, other medication, advice on how to manage symptoms etc* (SR11, 40–49, bisexual cisgender woman, currently going through menopause).

*Both of the NHS GPs I saw were men and neither seemed to know much about perimenopause other than I was ‘too young’ to be experiencing it. I felt dismissed and not listened to. I chose to pay to see a specialist and she was wonderful - listening carefully to all my symptoms and concerns, then prescribing HRT* (SR20, 40–49, bisexual cisgender woman, currently going through menopause).

*First GP dismissed me as depressed. Changed surgery when moved house and much better with immediate prescription for HRT* (SR52, 50–59, bisexual cisgender woman, currently going through menopause).

### Navigating minority sexualities

Concerns were raised by respondents about navigating their minority sexualities in relation to healthcare. For example,

*I haven't talked to my GP about menopause - I think this is partly related to my sexual orientation as I haven’t had a history of talking to them about issues related to sexual health, contraception etc. Although my GP surgery (where I lived when my symptoms were at their worst) was positive about supporting LGBTQ patients I had a few awkward experiences with nurses at smear tests when they asked me about sex, and I had to get them to clarify what they meant. So generally have talked more to friends etc about menopause than to medical practitioners* (SR2, 60–69, cisgender lesbian, previously went through menopause).

*Talking about some of the sexual issues with a male doctor not easy* (SR3, 50–59, cisgender lesbian, previously went through menopause).

*Having to ‘come out’ during GP consultation* (SR45, 60–69, cisgender lesbian, previously went through menopause).

*I found that when I’d declared being a lesbian there was more reluctance to provide sufficient treatment. I was even told by a consultant ‘the main impact of menopause on someone of your age is fertility but you do not need to worry about that as there will always be two wombs within your relationships’* (SR31, 18-29, cisgender lesbian, early menopause triggered by clinical condition, currently going through menopause).

### Heteronormative assumptions among providers

Concerns were raised about heteronormative assumptions (i.e. assuming patients are heterosexual) among healthcare providers. For example,

*I don’t think sexuality is taken into consideration for any female health issue and I think GP’s need to have awareness raised with regard to not assuming all women are straight and to perhaps to be more sensitive when discussing sexual issues to the fact that someone may be gay* (SR36, 50–59, gay cisgender woman, currently going through menopause).

*Different sexual experiences /different living arrangements - GPs can make heteronormative assumptions. Also menopause support groups are often very heteronormative* (SR6, 60–69, bisexual woman, currently going through menopause).

*My experience of menopause services (GP, NHS menopause clinic, private menopause clinic) is that they are all very heteronormative… Menopause doctors have tended to ask me ‘how sex is with my husband’… I already feel like a ‘freak’ as a disabled person. They do not entirely feel like safe spaces for me* (SR28, 40–49, bisexual cisgender woman, currently going through menopause).

*There was more silence about it in the past, but GP services are still heteronormative, meaning that lesbians and bisexual women’s reproductive health concerns are still not taken as seriously as those of heterosexual women* (SR2, 60–69, cisgender lesbian, previously went through menopause).

### Heterosexist information and guidance

Concerns were raised about heterosexist (heterosexuality-privileging) menopause information and guidance. For example,

*Heteronormative healthcare affected my treatment for cancer and my menopause… when I asked for help with sexual dysfunction the help that was offered was about heterosexual sex. My ‘husband’ would be referred to even though I’m not married* (SR26, 40–49, cisgender lesbian, currently going through menopause).

*If experiencing loss of libido, lots of the advice is aimed at straight women (*SR20, 40–49, bisexual cisgender woman, currently going through menopause).

*There is a big focus on penetration-oriented sex and there is zero information about how two women experiencing menopause together may have exacerbated problems or even if things like topical HRT can impact a partner not on HRT* (SR22, 50–59, cisgender lesbian, currently going through menopause).

*HRT labels don't specify risks to female partners* (SR33, 30–39, cisgender lesbian, previously went through menopause).

## Discussion

This study is based on a small non-representative sample and so its findings should be approached with caution. The SMC women in this study reported similar menopause symptoms to heterosexual cisgender (HC) women in other studies.^6,7,11^ They described higher rates of anxiety and depression, echoing previous research indicating SMC women have worse general mental health than HC women.^23^ The greater proportion of bisexual women reporting menopause-related mental health issues than the lesbians also echoes research indicating comparatively worse general mental health among bisexual women compared with lesbians.^14,24^

The SMC women share many menopause healthcare concerns with HC women regarding access to, and quality of, GP support; variable HRT prescribing; and quality of information. However, this is complicated for some SMC women by their respective minority sexual identities, barriers to disclosure, heteronormative/heterosexist GP microaggressions, and exclusionary menopause information. While dissatisfactions were most commonly reported by participants who had previously experienced the menopause – suggesting there had been recent improvements – the fact that a third of SMC women currently experiencing the menopause are also dissatisfied, suggests that there is still considerable room for improvement.

There are several ways to take these findings forward. Firstly, GPs need to be more willing and able to respond affirmatively to SMC women and address their healthcare needs appropriately. Secondly, GP menopause education should address issues specifically affecting SMC women. Thirdly, menopause patient information should both recognise issues affecting SMC women and include information specifically for them.^14,25^

Advocates and policy-makers must also address the needs of SMC women. The British Menopause Society’s ‘Vision for Menopause Care in the UK’ report makes no reference to them.^26^ Neither does the European Menopause and Andropause Society (EMAS) position statement on an essential menopause curriculum for healthcare professionals.^27^ Both require urgent revision to incorporate SMC women’s concerns. NICE guidance on menopause care^18^ should also be revised to address minority sexualities, for example, the impact of HRT on partners of women in same-sex relationships. Examples of other types of women’s healthcare information inclusive of SMC women are regrettably rare, this study echoing wider shortfalls. However, one example of healthcare information dedicated to SMC women is Public Health England’s online document ‘Cervical Screening for Lesbians and Bisexual Women’.^28^ It emphasises that ‘cervical screening is not just for heterosexual women’ and provides tailored guidance, including how to deal with GPs who mistakenly think only heterosexual women need screening. While this is to be celebrated, it would also be good to see it included in generic guidance for all women, to avoid ‘othering’ SMC women.

SMC women are legally, morally, and ethically entitled to equitable menopause care. All women deserve improved standards of menopause healthcare and support, and an effective inclusive response to their diverse menopause experiences.
